# Ferroptosis-Related lncRNA Signature Correlates with the Prognosis, Tumor Microenvironment, and Therapeutic Sensitivity of Esophageal Squamous Cell Carcinoma

**DOI:** 10.1155/2022/7465880

**Published:** 2022-07-16

**Authors:** Jiahao Zhu, Yutian Zhao, Gang Wu, Xiaojun Zhang, Qingqing Chen, Bo Yang, Xinwei Guo, Shengjun Ji, Ke Gu

**Affiliations:** ^1^Department of Radiotherapy and Oncology, The Affiliated Hospital of Jiangnan University, Wuxi, Jiangsu 214000, China; ^2^Department of Radiotherapy and Oncology, the Affiliated Suzhou Hospital of Nanjing Medical University, Gusu School, Nanjing Medical University, Suzhou, Jiangsu 215000, China; ^3^Department of Radiation Oncology, The Affiliated Taixing People's Hospital of Yangzhou University, Taizhou, Jiangsu 225300, China

## Abstract

Esophageal squamous cell carcinoma (ESCC) is the most prevalent form of esophageal cancer in China and is closely associated with malignant biological characteristics and poor survival. Ferroptosis is a newly discovered iron-dependent mode of cell death that plays an important role in the biological behavior of ESCC cells. The clinical significance of ferroptosis-related long noncoding RNAs (FRLs) in ESCC remains unknown and warrants further research. The current study obtained RNA sequencing profiles and corresponding clinical data from The Cancer Genome Atlas (TCGA) and Gene Expression Omnibus (GEO) databases, and FRLs were obtained through coexpression analysis. Consensus clustering was employed to divide the subjects into clusters, and immune-associated pathways were identified by functional analysis. The current study observed significant differences in the enrichment scores of immune cells among different clusters. Patients from TCGA-ESCC database were designated as the training cohort. A ten-FRL prediction signature was established using the least absolute shrinkage and selection operator Cox regression model and validated using the GEO cohort and our own independent validation database. Real-time quantitative polymerase chain reaction was used to verify the expression of the ten FRLs, and the ssGSEA analysis was employed to evaluate their function. In addition, the IMvigor database was used to assess the predictive value of the signature in terms of immunotherapeutic responses. Multivariate Cox and stratification analyses revealed that the ten-FRL signature was an independent predictor of the overall survival (OS). Patients with ESCC in the high-risk group displayed worse survival, a characteristic tumor immune microenvironment, and low immunotherapeutic benefits compared to those in the low-risk group. Collectively, the risk model established in this study could serve as a promising predictor of prognosis and immunotherapeutic response in patients with ESCC.

## 1. Introduction

Esophageal cancer is the eighth most prevalent type of cancer and sixth leading cause of cancer-related deaths worldwide. Regardless of the multidisciplinary approach employed in the management of esophageal cancer, including surgery, chemoradiotherapy, and immunotherapy, the five-year survival rate remains approximately 20% [1]. Esophageal squamous cell carcinoma (ESCC) is the main histological type of esophageal cancer, particularly in Eastern Asia [2]. The median survival of the majority of patients with ESCC is less than 10 months, owing to the fact that the disease is often diagnosed at an advanced stage, which poses a great challenge from a therapeutic perspective [3]. Hence, there is an urgent need to identify sensitive tumor factors and new specific biomarkers for precise diagnosis, individualized therapy, and prognosis prediction of patients with ESCC.

Ferroptosis is an iron-dependent programmed cell death induced by the accumulation of lipid-based reactive oxygen species and was originally proposed in 2012 [4, 5]. Recent studies have shown that ferroptosis is a significant regulatory mechanism involved in the growth and development of various malignancies. Consequently, ferroptosis-related long noncoding RNAs (FRLs) have been identified as effective biomarkers for predicting the prognosis of several malignancies, including lung [6], gastric [7], hepatocellular [8], and breast [9] carcinomas. Moreover, it may be considered that combination with agents that induce ferroptosis signaling may improve the antitumor efficacy, especially in clinical situations involving therapy-resistant carcinomas. Previous studies have reported that ferroptosis resistance leads to poor therapeutic efficacy and unfavorable prognosis in hepatocellular carcinoma [10, 11]. Ferroptosis plays a crucial role in ESCC development. A recent study reported that 5-aminolevulinic acid induces ferroptosis through the regulation of glutathione peroxidase 4 (GPX4) and heme oxygenase 1 (HMOX1) and exerts antitumor effects in ESCC [12]. Furthermore, another study reported that SLC7A11 inhibits ferroptosis and induces NRF2-associated radioresistance. These results indicate that SLC7A11 is a potential biomarker for ESCC [13]. The mechanism of FRLs in ESCC remains ambiguous, and further research is required to comprehend their biological functions.

Long noncoding RNAs (lncRNAs) are non-protein-coding RNAs with a molecular weight of >200 nucleotides [14]. Although lncRNAs do not encode proteins, they play significant roles in the stability and translation of cytoplasmic mRNAs and are involved in the regulation of signal transduction [15]. However, the literature includes few studies on the function of FRLs in ESCC. This scenario warrants the identification of FRLs that could serve as biomarkers and treatment targets.

A previous study demonstrated an in vivo interaction between the tumor immune microenvironment (TIME) and ferroptosis [16]. The TIME influences iron metabolism, and ferroptosis can augment the exposure of tumor antigens, thereby improving immunogenicity and promoting the efficacy of immunotherapy [17]. Recent clinical trials involving immunotherapy using immune checkpoint inhibitors (ICIs) have yielded a major breakthrough, and therapy has provided carcinoma patients with survival benefits [18]. Wang et al. reported that CD8+ T lymphocytes may induce ferroptosis in tumor cells in vivo through the downregulation of SLC7A11 and that ferroptosis-suppressed tumor cells are resistant to ICI therapy [19]. However, the current literature fails to elucidate the underlying mechanisms associated with ferroptosis and antitumor immunity due to the limited number of studies on this subject.

Tumor cells frequently exhibit abnormal expression of FRLs, which is associated with tumor development [20, 21]. However, the specific molecular mechanisms of FRLs in ESCC remain unknown and warrant further research. The current study is aimed at investigating the biological function of FRLs in ESCC using bioinformatics and identifying potential biomarkers and treatment targets to predict prognosis and improve treatment efficacy in ESCC patients. The present study used The Cancer Genome Atlas (TCGA) and Gene Expression Omnibus (GEO) databases to select FRLs associated with ESCC and identify ten lncRNAs with close correlation to the prognosis of ESCC. A prognostic multi-lncRNA signature was established using TCGA-ESCC database and validated by means of the GEO cohort using the least absolute shrinkage and selection operator (LASSO) Cox regression model. Real-time quantitative polymerase chain reaction (qRT-PCR) was used to verify the expression of ten FRLs. Finally, risk prediction tomography, functional enrichment analysis, and immune landscape analysis were performed to provide promising insights into the clinical outcomes, underlying mechanisms, and immunotherapy of ESCC.

## 2. Materials and Methods

### 2.1. Data Collection

The RNA sequencing dataset and corresponding clinical information regarding ESCC were obtained from TCGA (https://tcga-data.nci.nih.gov/tcga/) database. The expression profiles of GSE53624 and GSE53625, which contained 119 and 179 ESCC cases, respectively, were obtained from the GEO (https://www.ncbi.nlm.nih.gov/geo/) database. Moreover, the IMvigor210 cohort was obtained from the IMvigor210CoreBiologies in the R package, which comprised the expression files and clinical data of patients with advanced urothelial cancer who underwent ICI therapy, to assess the predictive value of the risk score in ICI therapy [22]. The clinical characteristics of the patients enrolled in this study are shown in Table S1 in the Supplementary Material.

### 2.2. Identification of Ferroptosis-Related lncRNAs

Data on ferroptosis-related genes (FRGs) were retrieved from previous studies [23]. FRLs were screened from FRGs by coexpression analysis of data obtained from TCGA-ESCC database [24]. The current study performed a correlation analysis of FRLs and FRGs to calculate Pearson's correlation coefficients. FRLs with absolute values of Pearson′s correlation coefficient > 0.30 and *P* value < 0.05 were selected. The Cytoscape software was used to construct and visualize a regulatory network between the selected FRLs and the corresponding FRGs.

### 2.3. Cluster Analysis Based on FRLs

In the present study, a consensus clustering analysis was performed using FRLs. First, the Cox regression analysis using the data obtained from TCGA-ESCC database was conducted to identify the candidate FRLs with close association to the overall survival (OS) (*P* < 0.05). Subsequently, the FRLs were extracted for clustering analysis. The “ConsensusClusterPlus” R program was used to implement clustering and the best cluster number was chosen as the value of coexistence correlation coefficient, *K*. The OS of the different subgroups was compared using the Kaplan–Meier survival curve.

### 2.4. Differential Expression and Functional Enrichment Analyses

The “limma” R package was applied to identify the significant differentially expressed mRNAs among different clusters with a false discovery rate < 0.05 and logFC > 1 in TCGA cohort. Venn diagrams were created by means of VENNY 2.1.0 (http://bioinfogp.cnb.csic.es/tools/venny/index.html) to identify common significant differentially expressed mRNAs between clusters. The PD-L1 expression in the different subgroups was compared, and a correlation analysis between PD-L1 expression and FRLs was performed. Moreover, Gene Ontology (GO) and Kyoto Encyclopedia of Genes and Genomes (KEGG) analyses of common significantly differentially expressed mRNAs between clusters were performed using the R package “clusterProfiler.” Potential biological processes, molecular functions, cellular components, and pathways associated with these mRNAs were explored.

### 2.5. Analysis of Immune Infiltration between Subgroups

The StromalScore, ImmuneScore, and ESTIMATEScore in the different ESCC clusters were calculated using the “ESTIMATE” R package. Two algorithms were used to quantify the immune cells and compare the abundance differences between these immune cells in different clusters. The infiltrating scores of ten immune cells were evaluated with the “MCPcounter” R package, and 28 immune cells were determined using single-sample gene set enrichment analysis (ssGSEA) with the “GSVA” R package [25]. Statistical significance was set at *P* < 0.05.

### 2.6. Identification and Validation of the Prognostic FRL Signature

The FRLs that displayed significance (*P* < 0.05) in both the Kaplan–Meier and univariate Cox analyses for OS were selected as the potential prognostic genes and then extracted into the LASSO analysis with the “glmnet” R package. Candidate FRLs were obtained when the best penalty parameter, lambda, was achieved in the training cohort [26]. The risk score was computed using the normalized gene expression level and the corresponding regression coefficient as follows: risk score = sum (gene expression level corresponding coefficient). Subsequently, patients were divided into high- and low-risk groups according to their respective median risk scores. Time-dependent ROC curve analysis was performed to assess the prognostic predictive value of the model with the “risksetROC” R package. The difference in survival between the two groups was assessed using the Kaplan–Meier method. The same formula and statistical analyses were used to analyze the external validation database of the GSE53624 cohort to test the stability of the model developed in the current study. Survival differences between high- and low-expression candidate FRLs are shown with the Kaplan–Meier curves. Differences in clinical variables between the high- and low-risk groups were compared using the Wilcoxon test. Kaplan–Meier curves were used to compare survival differences between the high- and low-risk groups stratified by clinical characteristics.

### 2.7. Tissue Sample Collection and FRL Expression Detection

A total of 35 human ESCC tissues were obtained from the Affiliated Hospital of Jiangnan University (Wuxi, Jiangsu, China) and the Suzhou Municipal Hospital (Suzhou, Jiangsu, China) from 2018 to 2021. The previously collected tissue samples were stored and transported at -80°C. The study was approved by the Affiliated Hospital of Jiangnan University and the Suzhou Municipal Hospital for Biomedical Research Ethics Committee, and these patients signed the informed consent. The clinical characteristics of these patients enrolled in this study are shown in Table S1.

The RNA was extracted from 35 ESCC tissues, TRIzol (Invitrogen, Carlsbad, CA, USA), according to the reagent instructions, and purified with the RNeasy Mini reagent (Qiagen, Valencia, CA, USA). The amount and quality of RNA were evaluated by spectrophotometer (ND-1000, Nano Drop Technologies, Wilmington, De, USA), and the integrity of RNA was evaluated by gel electrophoresis. We used Arraystar Human lncRNA V3.0 chip to analyze RNA samples. The chip could detect more than 30,000 lncRNAs. We removed rRNA from the total RNA and obtained mRNA (mRNA-ONLYTM Eukaryotic mRNA Isolation Kit, Epicentre). Oligo (dT) and random primers were mixed to amplify each sample and transcribed into fluorescent cRNAs, which were hybridized with human lncRNA chip. Agilent chip scanner (Agilent p/n G2565BA) scanned the chip after cleaning the slide, and the chip diagram was obtained by the Agilent Feature Extraction software (v11.0.1.1). After reading, the original data were obtained, using the Gene Spring GX v12.1 software (Agilent Technologies) for raw data standardization and subsequent data processing. After data standardization, select high-quality probes (if more than 17 of 35 samples were labeled Marginal or Present) for further analysis.

### 2.8. Validation of Expression of Prognostic FRLs

Human ESCC cells (KYSE-150 and ECA-109) and normal esophageal epithelial cells (HET-1A) were obtained from the Shanghai Institute of Cell Biology (Shanghai, China). The cells were cultured in an RPMI 1640 medium (Gibco) supplemented with 10% fetal bovine serum under a humidified atmosphere of 37°C and 5% CO_2_. HET-1A cells were cultured in serum-free LHC-9 medium. Cellular RNA was extracted using RNeasy kits (Qiagen, Hilden, Germany) and quantified using Nanodrop (Thermo Fisher). The Quantitect Reverse Transcription Kit (QIAGEN) was used to reverse-transcribe total RNA for the synthesis of cDNA, in accordance with the manufacturer's instructions. qRT-PCR was used to determine the relative expression levels of lncRNAs in triplicate using a StepOnePlus Real-Time PCR System (Applied Biosystems). GAPDH was used as an internal control. The relative expression of each lncRNA was estimated by the 2^−*ΔΔ*Ct^ method. Primer sequences are listed in the supplementary file (see Table S2 in the Supplementary Material). In addition, a paired *t*-test was performed to assess the difference in the expression levels of prognosis-related FRLs in ESCC and corresponding paracancerous tissues in the GSE53625 database.

### 2.9. Nomogram Development and Evaluation of Predictive Performance

The current study performed univariate and multivariate Cox regression analyses to identify independent prognostic factors based on the patient's clinical information and risk score, providing clinicians with a more accurate quantitative method for the prediction of OS in patients with ESCC. We tested the proportional hazards assumption by Schoenfeld residuals with the “RMS” R package. Subsequently, a nomogram was created using the survival rate and “RMS” R package, and a calibration curve was formulated to assess the consistency between actual and predicted survival rates. Discrimination was used to evaluate the predictive ability of the nomograms. We used likelihood ratio tests to compare Cox proportional hazard models.

### 2.10. Analysis of Biological Properties and Pathways Related to the Signatures

In the GSE53624 database, the gene set enrichment analysis (GSEA) was used to analyze the potential biological activities and signal transduction pathways associated with FRLs in the high- and low-risk categories of patients with ESCC. The GSEA software was used. |NES| > 1 and *P* < 0.05 were considered significant.

### 2.11. Immunogenomic Landscape Analyses between High-Risk and Low-Risk Groups

A comprehensive analysis of the immune cells and pathways was conducted using ssGSEA between the high- and low-risk groups in the training cohort. Immune checkpoint gene expression levels were compared between high-risk and low-risk groups to explore the relationship between the risk score and immune checkpoints. A two-sample Wilcoxon test was used to compare differences between the two groups.

### 2.12. Analysis of FRL Signatures in Immunotherapy

The data obtained from the IMvigor210 cohort were analyzed to validate the predictive power of the risk score model for immunotherapy. The present study evaluated the differences between the high- and low-risk groups with regard to survival and treatment response. The ROC analysis was performed to assess the prognostic ability of the risk model. In addition, we performed a correlation analysis of the risk score and infiltrating level of immune cells, neoantigen (NEO), and tumor mutation burden (TMB).

### 2.13. Statistical Analysis

The present study employed Student's *t*-test to identify the differentially expressed FRGs among different clusters and to analyze the differences between high- and low-risk groups with regard to ImmuneScore, StromalScore, and ESTIMATEScore. Characteristics pertaining to the risk groups were compared using chi-square or Fisher's exact tests. The Mann–Whitney test was used to assess the difference between the high- and low-risk groups with regard to the ssGSEA scores of immune cells or pathways. All statistical analyses were performed using R version 3.6.3 or GraphPad Prism version 8.0. All *P* values were two-tailed. In the current study, a *P* value < 0.05 was considered statistically significant.

## 3. Results

**Figure 1:**
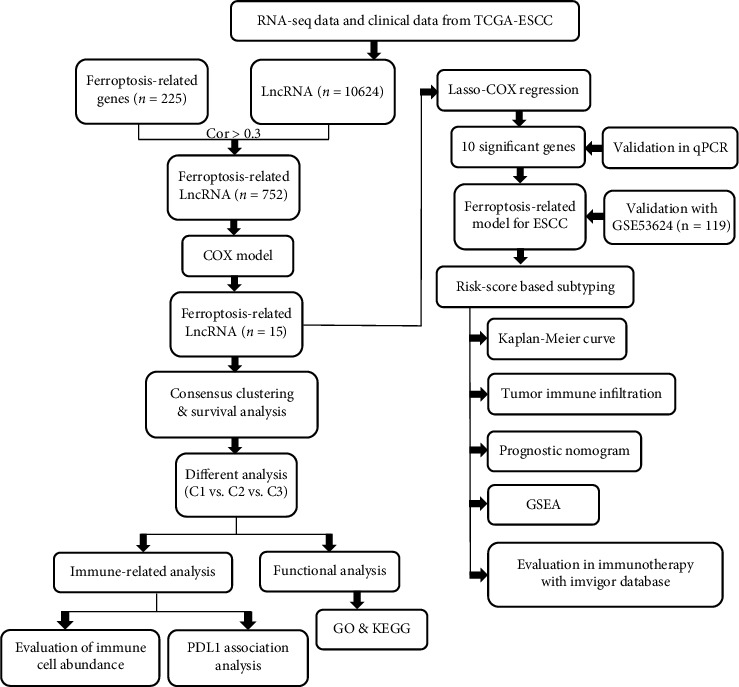
Flowchart of the data collection and analysis.

The detailed workflow of this study is shown in Figure 1. The current study involved 81 ESCC patients from TCGA-ESCC database, 119 ESCC patients from the GSE53624 cohort, 179 cases from the GSE53625 cohort, and 348 cases from the IMvigor210 cohort. TCGA-ESCC database was considered as the training cohort, and the remaining databases were considered validation cohorts with reference to the prognosis predictive model, expression of FRLs, and sensitivity to immunotherapy.

### 3.1. Identification of FRLs Associated with the Prognosis in ESCC Patients

In this study, extracted 10,624 lncRNAs using RNA sequencing data from TCGA-ESCC cohort, and 225 ferroptosis-associated genes were retrieved from the ferroptosis database. The FRLs were identified using Pearson's correlation analysis (correlation coefficient > 0.3 and *P* < 0.001). Subsequently, 752 FRLs were selected (see Figure S1).

### 3.2. Classification of ESCC on the Basis of FRLs

**Figure 2:**
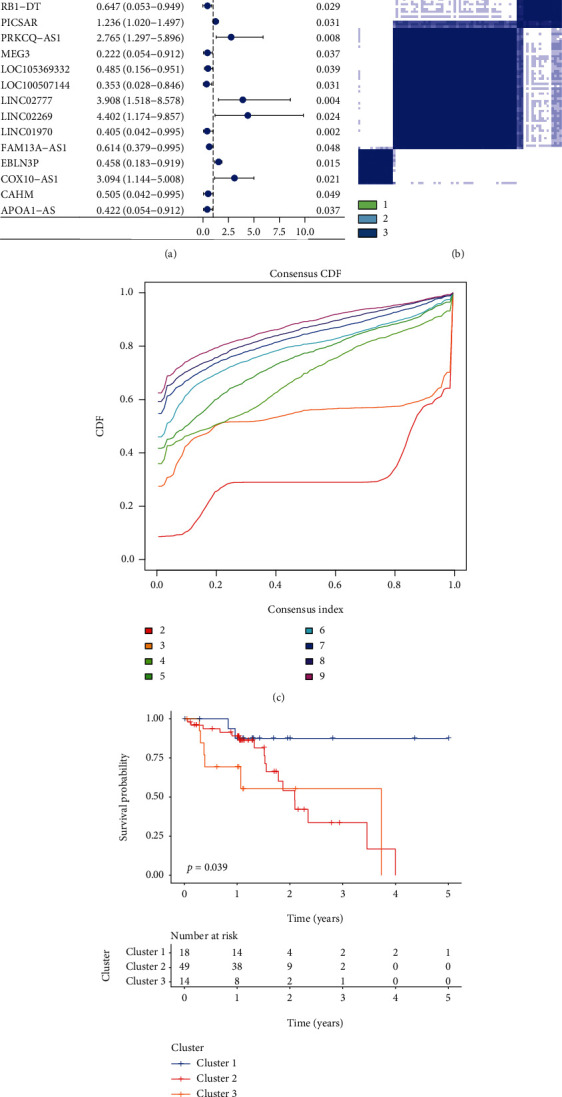
Identification of ESCC subclasses using consensus clustering. (a) Univariate Cox regression analysis. Forest plot of 15 significant FRLs associated with the overall survival in TCGA-ESCC cohort. (b) The patients were divided into clusters 1, 2, and 3 on the basis of 15 FRLs. (c) Empirical cumulative distribution function plot displaying consensus distributions for each *k*. (d) Survival analysis of the patients in clusters 1, 2, and 3 in TCGA cohort.

In the present study, 15 FRLs were selected on the basis of their significant prognostic value with regard to OS (*P* < 0.05) and subjected to a consensus clustering analysis (see Figure 2(a)). Samples from TCGA-ESCC database were divided into three clusters using the “ConsensusClusterPlus” R package. The optimal *k* value was determined using the correlation coefficient. Subsequently, the optimal total cluster number was set to *k* = 3 (with the three subclasses designated as clusters 1, 2, and 3; see Figures 2(b) and 2(c)). The current study observed a significant difference among the three clusters with regard to OS in TCGA cohort (*P* = 0.039; see Figure 2(d)).

### 3.3. Differential Expression among Clusters and Functional Enrichment Analysis

**Figure 3:**
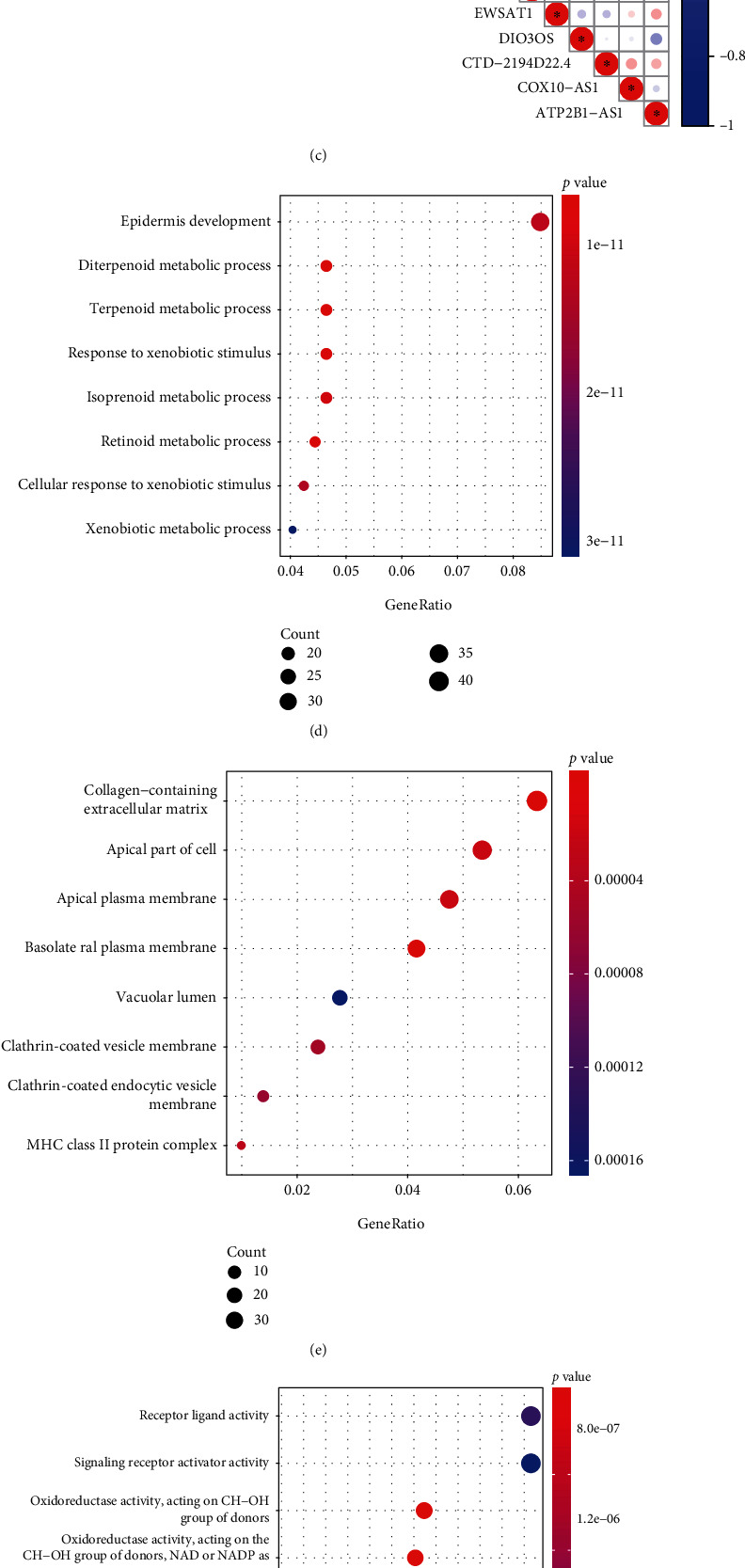
(a) Identification of differentially expressed mRNAs among the clusters 1, 2, and 3 in TCGA-ESCC cohort. (b) Comparison of the expression of PD-L1 among the three clusters. (c) The heat map shows the association between PD-L1 and differentially expressed mRNAs among clusters. The Gene Ontology annotation of differentially expressed genes. The significantly associated canonical pathways are shown as follows: (d) top eight related biological processes (BP), (e) cell component (CC), and (f) molecular function (MF). Results of pathway enrichment for differentially expressed genes. (g) The top eight significantly altered canonical pathways are shown. Adjusted *p* values: ^∗^*P* < 0.05. NS (no significance): *P* > 0.05.

The “limma” R package with FDR < 0.05 and logFC > 1.0 was used to identify 29 mRNAs as significant differentially expressed genes (DEGs) among the three clusters, as shown in Figure 3(a). The volcano plot shows the fold change and statistical significance of mRNA expression among the three clusters (see Figures S2A-S2C in the Supplementary Material). PD-L1 expression in Cluster 1 was higher than that in Cluster 2 (*P* < 0.05) (see Figure 3(b)). The relationship between PD-L1 expression and the 29 DEGs is shown in Figure 3(c). The GO functional analysis was conducted based on the DEGs. The top eight biological processes-associated, cell component-associated, and molecular function-associated categories are shown in Figures 3(d)–3(f). The top eight categories of KEGG functional analyses are shown in Figure 3(g).

### 3.4. Immune Infiltration between Subtypes

**Figure 4:**
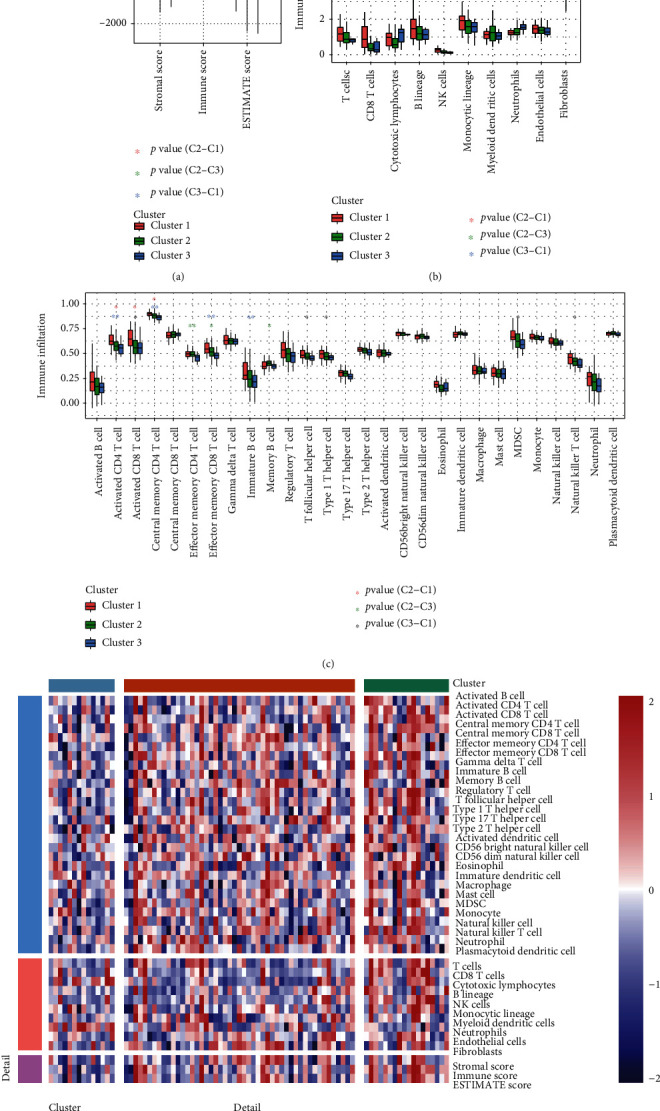
(a) Comparison of StromalScore, ImmuneScore, and ESTIMATEScore among the three clusters in TCGA-ESCC cohort. (b) Evaluation of the infiltrating scores of ten immune cells among the three clusters in TCGA-ESCC cohort. (c) Comparison of the ssGSEA scores among the three clusters in TCGA cohort. (d) The heat map denotes the immune scores pertaining to the three clusters. Adjusted *P* values: ^∗^*P* < 0.05; ^∗∗^*P* < 0.01; ^∗∗∗^*P* < 0.001.

StromalScore, ImmuneScore, and ESTIMATEScore were computed using the “ESTIMATE” R package. In the current study, the ImmuneScore was significantly higher in Cluster 1 than in Cluster 2 (*P* < 0.05), whereas the ImmuneScore and ESTIMATEScore were higher in Cluster 2 than in Cluster 3 (*P* < 0.01) (see Figure 4(a)). In addition, ten immune cell scores were evaluated using the “MCPcounter” R package, and the results revealed that the immune cell scores of CD8+ T cells and natural killer cells were higher in Cluster 1 than in Cluster 2 (*P* < 0.05). Immune cell scores of cytotoxic lymphocytes and neutrophils were higher in Cluster 3 than in Cluster 2 (*P* < 0.01). Immune cell scores for T cells, B lineage, and NK cells were higher in Cluster 1 than in Cluster 3 (*P* < 0.05), while the scores for neutrophils were higher in Cluster 3 than in Cluster 1 (*P* < 0.001) (see Figure 4(b)). The results of ssGSEA analysis showed that the immune scores of activated CD4 T cells, activated CD8 T cells, and central memory CD4 T cells were significantly higher in Cluster 1 than in Cluster 2 (*P* < 0.05). The immune scores of effector memory CD4 T cells, effector memory CD8 T cells, and memory B cells were higher in Cluster 2 than in Cluster 3 (*P* < 0.05). The immune scores for activated CD4 T cells, activated CD8 T cells, central memory CD4 T cells, effector memory CD8 T cells, immature B cells, T follicular helper cells, type 1 T helper cells, MDSCs, and natural killer T cells were higher in Cluster 1 than in Cluster 3 (*P* < 0.05) (see Figure 4(c)). A comparison of the three immune scores pertaining to the molecular subtypes is shown in Figure 4(d), using a heat map.

### 3.5. Identification of the Prognostic FRL Signature

In the current study, the 15 FRL prognostic genes were subjected to OS-based LASSO Cox regression model analysis (see Figure S3A). The regression model attained optimal ability after the identification of ten prognostic lncRNAs: SNHG29, RB1-DT, MEG3, LOC100507144, LINC02269, LINC01970, FAM13A-AS1, EBLN3P, CAHM, and APOA1-AS (see Figure S3B). Kaplan–Meier curves of these lncRNAs revealed a significant association with OS in TCGA-ESCC cohort (see Figure S4 in the Supplementary Material).

### 3.6. A Ferroptosis-Related Prognosis Model Construction in TCGA Cohort

The following formula was used to generate a hazard model through the linear mixing of ten FRLs weighted by their coefficients from the multivariate Cox analysis:

Risk score = (*E*_SNHG29_×−0.71) + (*E*_RB1−DT_×−0.84) + (*E*_MEG3_×−1.02) + (*E*_LOC100507144_ × 0.15) + (*E*_LINC02269_ × 0.45) + (*E*_LINC01970_×−0.71) + (*E*_FAM13A−AS1_×−0.34) + (*E*_EBLN3P_ × 0.36) + (*E*_CAHM_×−0.64) + (*E*_APOA1−AS_×−0.68).

*E*_SNHG29_ denotes the expression value of *SNHG29*, and this applies to the rest of the acronyms in this formula.

**Figure 5:**
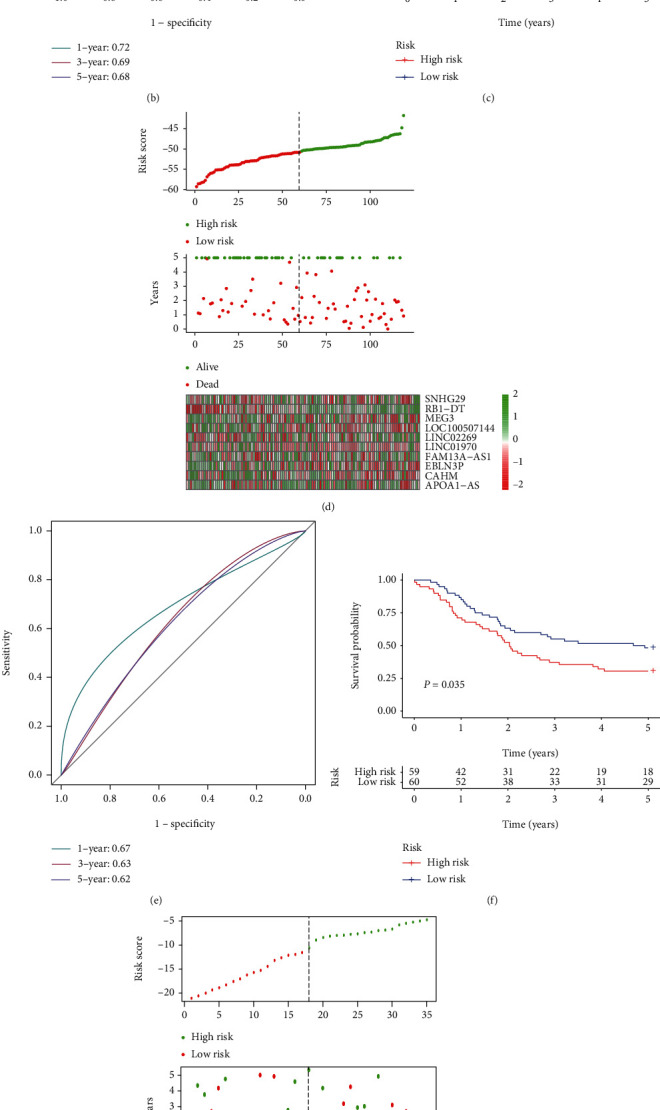
Construction of the prognostic prediction model and model validation. (a, d, g) Risk score (top), overall survival (middle) of the patients, and expression profiles of the 10 FRLs (bottom) in TCGA (training), GSE53624 (external validation) datasets, and independent validation cohort. (b, e, h) The areas under the time-dependent ROC curves verified the prognostic performance of the risk scores in TCGA, GSE53624 datasets, and independent validation cohort. (c, f, i) Kaplan–Meier curves for overall survival in the high- and low-risk groups in TCGA, GSE53624 datasets, and independent validation cohort.

The aforementioned approach was used to compute the risk score for each sample. The patients in TCGA cohort were divided into high-risk (*n* = 40) and low-risk (*n* = 41) groups on the basis of the optimum cutoff value obtained by means of the “survminer” R package. The risk scores, OS of patients, and expression profiles of the ten FRLs are presented in Figure 5(a). The ROC analysis results are shown in Figure 5(b). The areas under the ROC curves for the one-, three-, and five-year duration were 0.724, 0.693, and 0.682, respectively. The high-risk group displayed worse OS than the low-risk group (*P* = 0.009), as shown by the Kaplan–Meier curves in Figure 5(c).

### 3.7. Validation of the Ten-Ferroptosis-lncRNA Signature Using the Test Dataset

The predictive power of the model was evaluated using the test dataset from the GSE53624 cohort (*n* = 119; 59 samples in the high-risk group and 60 in the low-risk group) using the same formula. The risk scores, OS of the patients, and expression profiles of FRLs are shown in Figure 5(d). The areas under the time-dependent ROC curves pertaining to the one-, three-, and five-year duration were 0.671, 0.634, and 0.619, respectively (see Figure 5(e)). Patients in the high-risk group displayed a worse survival rate than those in the low-risk group, which is concurrent with previous findings (see Figure 5(f)).

We further access the model with the independent validation cohort collected form our own institution (*n* = 35; 17 samples in the high-risk group and 18 samples in the low-risk group). The risk scores, OS of the patients, and expression profiles of the FRLs are shown in Figure 5(g). The areas under the time-dependent ROC curves pertaining to the one-, three-, and five-year duration were 0.632 and 0.711, respectively (Figure 5(h)). The high-risk group displayed a strong tendency of worse OS, though it did not reach the statistical difference (*P* = 0.091), as shown by the Kaplan–Meier curves in Figure 5(i).

### 3.8. Correlation of the Prognostic Risk Score with Pathological Features

The current study did not observe any significant differences in risk score with reference to sex, age, grade, and TNM stage of the disease (all *P* > 0.05) (see Figures S5A-S5D in the Supplementary Material). However, the risk score in Cluster 2 was higher than that in Cluster 1 (*P* = 0.035) (see Figure S5E in the Supplementary Material).

### 3.9. Survival Analysis Using Prognostic Risk Scores in Subgroups

The Kaplan–Meier analysis of the group of male patients (*P* = 0.012) with age ≤ 65 years (P = 0.041), grades 3–4 (*P* = 0.013), and stage III or IV at the time of diagnosis (*P* = 0.038) revealed that the high-risk group exhibited worse OS than the low-risk group (see Figures S6A-S6D in the Supplementary Material).

### 3.10. Univariate and Multivariate Cox Analyses of Prognostic Risk Scores and Individualized Prognostic Prediction Models

**Figure 6:**
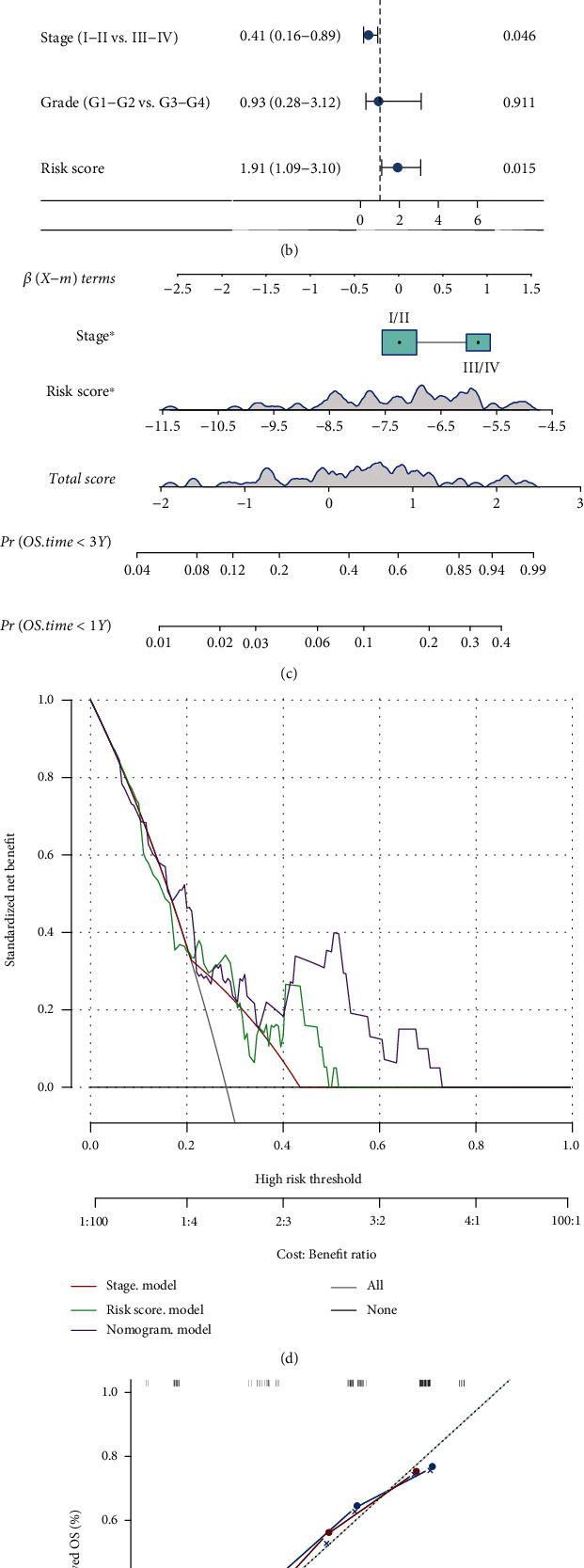
(a, b) Results of the univariate and multivariate Cox regression analyses of overall survival in TCGA cohort. (c) A nomogram of the ESCC cohort was used to predict the overall survival. (d) A decision curve analysis was used to compare the clinical efficacy of nomography with risk score and TNM stage, based on the threshold probability. (e) Calibration maps were used to predict one- and three-year survival rates.

TCGA-ESCC database was used to perform univariate and multivariate Cox regression analyses. Univariate Cox regression analysis revealed that risk scores and TNM stage were closely related to OS (*P* < 0.10) (see Figure 6(a)). Multivariate Cox regression analysis revealed that risk scores and TNM stage were independent predictors of OS in patients with ESCC (*P* < 0.05) (see Figure 6(b)). A global test of the Schoenfeld residuals for the nomogram model showed a *P* value of 0.287, and the *P* values of the Schoenfeld residual test for stage and risk score were 0.327 and 0.270, respectively (all *P* > 0.05) (see Figures S7A–S7B in the Supplementary Material).

A nomogram was established based on TNM stage and risk score through the synthesis of ten-FRL signatures to predict the probability of one- and three-year OS. Several factors were evaluated on the basis of the proportion of contribution to the death risk, as shown in Figure 6(c). The nomogram was an excellent predictive model, which was superior to the risk score or TNM stage alone (see Figure 6(d)). Furthermore, the calibration curve demonstrated a high correlation between the predicted and actual OS rates (see Figure 6(e)). A likelihood ratio test was applied to compare the nomogram model, including stage and risk score, with the stage model or risk score model. The resulting *P* values were 0.005 and 0.041, respectively, indicating that combining the stage and risk scores significantly improved the model fit (see Table S3 in the Supplementary Material).

### 3.11. Validation of Expression of the Prognostic FRLs

**Figure 7:**
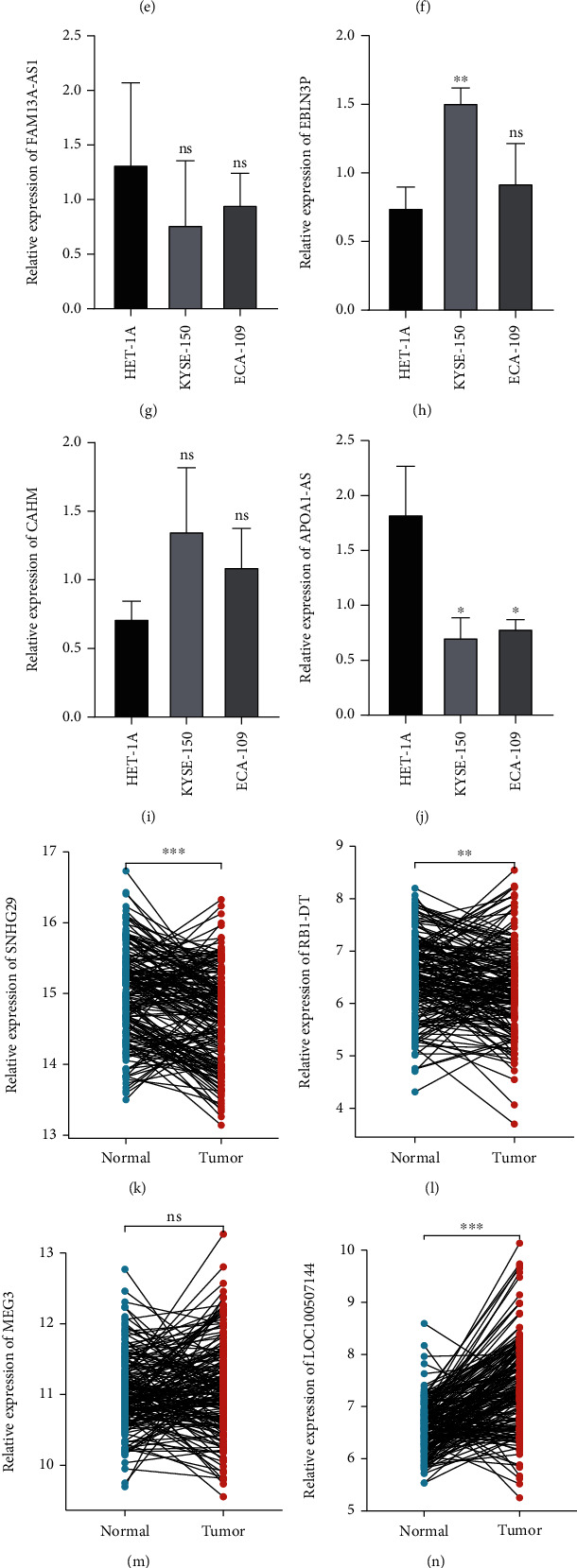
(a–j) Expression levels of SNHG29, RB1-DT, MEG3, LOC100507144, LINC02269, LINC01970, FAM13A-AS1, EBLN3P, CAHM, and APOA1-AS were validated in ESCC and normal esophageal epithelial cells. (k–t) Validation of the expression levels of the ten FRLs in the paracancerous (*n* = 179) and ESCC tissue samples (*n* = 179) by paired analysis in the GSE53625 cohort. Significant differences were defined as ^∗^*P* < 0.05, ^∗∗^*P* < 0.01, and ^∗∗∗^*P* < 0.001.

The qRT-PCR results revealed that the expression of LOC100507144, LINC02269, and EBLN3P was upregulated, whereas the expression of MEG3, SNHG29, RB1-DT, LINC01970, FAM13A-AS1, and APOA1-AS was downregulated in ESCC tissues compared to that in normal tissues. However, the current study did not observe any significant differences in the expression of FAM13A-AS1 and CAHM (see Figures 7(a)–7(j)). In addition, the expression of these 10 FRLs in the GSE53625 cohort was assessed using a paired t-test (see Figures 7(k)–7(t)). The results of qRT-PCR and bioinformatics analyses revealed similar trends in expression.

### 3.12. GSEA and Immune Infiltration between High- and Low-Risk Groups

The current study identified 54 pathways associated with ferroptosis-related lncRNAs (*P* < 0.05), and five representative upregulated signals were selected, including the VEGF signaling pathway, IL-17 signaling pathway, ErbB signaling pathway in the high-risk group, and Wnt signaling pathway and ferroptosis signaling pathway in the low-risk group (see Figures S8A–S8E in the Supplementary Material).

**Figure 8:**
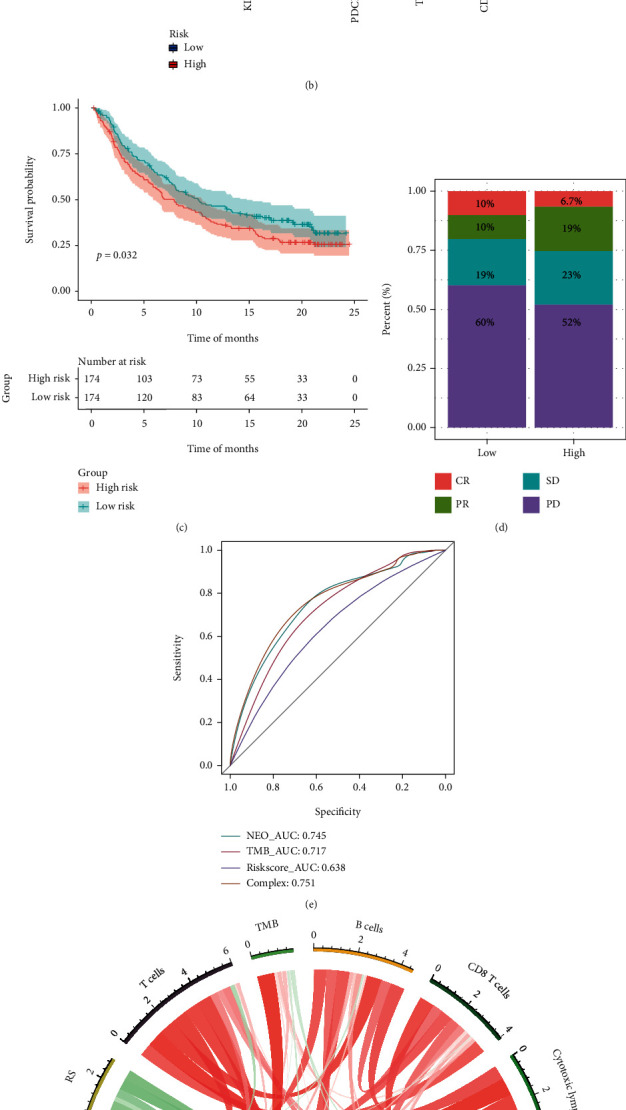
(a) Immune infiltration analysis of the high- and low-risk groups in boxplots. (b) Expression of immune checkpoints between high- and low-risk groups. (c) Kaplan–Meier curves of the overall survival in the high- and low-risk groups with IMvigor (external validation) datasets. (d) Comparison of efficacy of immunotherapy in the high- and low-risk groups in the IMvigor cohort. (e) The areas under the ROC curves verified the prognostic performance of the NEO, TMB, risk scores, and a combination of the above in the IMvigor (external validation) datasets. (f) Analysis of the association between risk score and NEO, TMB, and immune infiltration cells.

Comparative analysis of the immune cells and pathways demonstrated the differences between high- and low-risk groups with regard to CD8+ T cells, chemokine receptor (CCR), checkpoint, dendritic cells (DCs), macrophages, mast cells, MHC class I, neutrophils, and type II IFN response (*P* < 0.05) (see Figure 8(a)). The present study performed further research regarding the difference between the two groups in the expression of immune checkpoints on account of the significance of checkpoint-based immunotherapy (see Figure 8(b)).

### 3.13. FRL Signatures in Immunotherapy

Data pertaining to patients with advanced urothelial cancer who underwent ICI therapy with anti-PD-L1 (IMvigor210 cohort) were used to identify the predictive value of the ten-FRL-signature in immunotherapy treatment. The subjects were classified into high- and low-risk score subtypes based on their signature. Kaplan–Meier curves demonstrated that patients with higher risk scores displayed poorer OS than those in the low-risk group (*P* = 0.032) (see Figure 8(c)). A higher complete response rate was observed in the low-risk group (*P* = 0.06) (see Figure 8(d)). The areas under the ROC curve for NEO, TMB, risk score, and a combination of the above were 0.745, 0.717, 0.638, and 0.751, respectively (see Figure 8(e)). The correlation analysis of the risk score and infiltrating levels of immune cells, NEO, and TMB is shown in Figure 8(f).

## 4. Discussion

ESCC is one of the most common forms of malignancy and is prevalent worldwide, especially in East Asia. In clinical practice, the prognosis of patients with ESCC is assessed according to the pathological stage of the disease. However, the treatment efficacy differs among patients with identical pathological stages and grades of the disease. In view of the frequent application of clinical tumor sequencing, which is becoming more common, biomarkers display promising potential with reference to tumor detection and prognosis prediction.

For patients with early ESCC, several biomarkers, including PTEN, STMN1, and TNFAIP8, were screened and showed a good ability to predict lymph node metastasis after surgery [27–29]. In locally advanced ESCC, chemoradiotherapy plays an important role in the treatment of ESCC, regardless of neoadjuvant, postoperative adjuvant, or radical treatment. Therefore, identifying reliable predictive biomarkers for chemoradiation response is necessary. A meta-analysis showed that TP53 allele loss is closely related to poor response to chemotherapy [30]. The single nucleotide polymorphism state of ERCC1, a DNA damage repair gene, was reported to be a useful predictive genetic biomarker for chemoradiation treatment outcomes in ESCC [31]. A recent study reported that a seven-FRG signature had a higher predictive value for ESCC than TNM stage alone [32]. In terms of proteomics biomarkers, CEA, SCC, and carbohydrate antigen 72-4 (CA 72-4) have been applied in the cancer management and provides treatment guidance for clinicians [33, 34]. Kim et al. developed new scores for ES treatment response prediction. The tumor-derived fraction of cell-free DNA (cfDNA) profiles was examined by whole genome sequencing in blood samples of 30 ES cases, and low scores were found to have a significant association with better chemoradiotherapy response [35]. Noncoding RNAs, including miRNAs and lncRNAs, play important roles in various biological processes, such as tumor proliferation [36], hypoxia [37], metabolism [6], methylation [38], apoptosis [39], and autophagy [40], and have been investigated as potential biomarkers or therapeutic targets in ESCC. A study by Chen et al. found that high levels of miR-133a/b were associated with significantly long OS in ESCC and were an independent prognostic factor for these patients [36]. A nine-autophagy-related lncRNA signature established by Shi et al. showed a favorable treatment outcome prediction value for ESCC [40]. Several recent studies have reported that FRLs affect the development and progression of solid tumors by serving as competing endogenous RNAs, such as OIP5-AS1 in prostate cancer and MT1DP in non-small-cell lung cancer [41, 42]. Additionally, literature has reported that regulation of the process of cell death by FRLs improves therapeutic outcomes in tumors [43]. Thus, potential lncRNAs warrant further research, owing to the fact that FRLs play an important role in malignancies, and the current knowledge regarding the depth mechanism is limited.

The current study selected ten prognosis-related FRLs and a signature that combined these lncRNAs displayed a favorable survival prediction value in ESCC. A recent study demonstrated that SNHG29 (small nucleolar RNA host gene 29) plays an important role in the invasion, migration, and epithelial-to-mesenchymal transition of laryngeal cancer, suggesting that SNHG29 is a potential therapeutic target [44]. In addition, another study reported that the inhibition of SNHG29 could improve antitumor immunity through the activation of the YAP pathway in colorectal cancer [45]. Interventions involving SNHG29 may provide a synergistic effect and promote the efficacy of immunotherapy in the management of CRC. Moreover, MEG3 (maternally expressed gene 3), which is expressed at a low level in ESCC, has been reported to inhibit the proliferation, migration, and apoptosis of ESCC cells in vitro and tumor development in vivo [46, 47]. In addition, a previous study reported that MEG3 regulates T cell differentiation and contributes to immune escape in esophageal cancer [48]. Furthermore, FAM13A-AS1 (FAM13A antisense RNA 1), which is associated with autophagy, is included in the lncRNA signature to predict the prognosis of thyroid cancer and bladder urothelial carcinoma [49, 50]. Xu et al. reported that EBLN3P (endogenous bornavirus-like nucleoprotein 3, pseudogene) promoted the development of colorectal cancer by regulating the expression of UHMK1 [51]. It has been reported that DNMT1 suppresses colon adenocarcinoma hypermethylation (CAHM) and promotes tumor progression through the SPAK/JNK pathway in glioma [52]. It is worth mentioning that the current study is the first to report the association between ESCC prognosis and RB1-DT (RB1 divergent transcript), LOC100507144, LINC02269, LINC01970, and APOA1-AS (APOA1 antisense RNA). In summary, three of the FRLs (LOC100507144, LINC02269, and EBLN3P) in the prognostic model were reported to be upregulated in ESCC, in contrast to the remaining seven lncRNAs (SNHG29, RB1-DT, MEG3, LINC01970, FAM13A-AS1, CAHM, and APOA1-AS). Nevertheless, the underlying mechanism, that is, whether these lncRNAs influence ferroptosis and affect the prognosis of patients with ESCC, remains ambiguous owing to the limited number of previous studies regarding the same.

Further investigation revealed that FRLs could dichotomize ESCC patients into different risk groups for discernment of OS. Functional analyses of FRLs in these subgroups revealed significant differences in immune-related pathways, including the MHC class II protein complex and IL17 signaling pathways. These results warrant further research on the underlying mechanisms associated with immunity and ferroptosis in ESCC. The immune cell landscape in the TIME showed that the number of mast cells, macrophages, and neutrophils was significantly higher, and the number of CD8+ T cells was higher in the high-risk group than in the low-risk group. Moreover, the current study observed differences in ICIs, such as B and T lymphocyte associated (BTLA), CD200, CD48, CD27, and CD28. These differences imply a sophisticated relationship between ferroptosis and immunity.

Several studies have shown that increased numbers of mast cells, macrophages, and neutrophils are associated with poor prognosis in certain solid tumors, which is consistent with the observations in the current study [53]. The present study observed that the proportion of CD8+ T cells was high in ESCC patients with a low risk. Studies have reported that mast cells secrete angiogenic factors and proteases, which stimulate angiogenesis and breakdown of the extracellular matrix, thereby contributing to the invasion of tumor cells [54]. A high density of mast cells in the tumor microenvironment is a predictor of poor survival in patients with ESCC and is closely associated with tumor angiogenesis and metastasis [55]. Interestingly, the number of activated macrophages and CD8+ T cells in esophageal tissue is positively correlated with the level of IL-17-producing mast cells, which indicates favorable clinical outcomes [56]. The function of mast cells in the tumor microenvironment of esophageal carcinoma remains unclear, and elucidation of the specific underlying mechanism requires further investigation. M2 macrophages, which account for the majority of tumor-associated macrophages in esophageal carcinoma tissues, are strongly associated with angiogenesis and tumor invasion, and the presence of these macrophages predicts unfavorable outcomes concerning survival [57]. Macrophages can also be recruited into the TME to promote immunocyte infiltration via the PD-1/PD-L2 pathway, which may open a new avenue for anticancer immunotherapy in the management of ESCC [58].

Recently, a multidisciplinary treatment strategy for ESCC has been developed. However, the prognosis for this malignancy remains poor. The outcomes of clinical trials in ESCC, including ESCORT, KEYNOTE-181, and ATTRACTION-03, imply that the inclusion of immunotherapy in the management protocol could improve survival rates [59–61]. ICIs have shown promising prospects in the treatment of ESCC. However, PD1/PDL-1 and CTL-4 remain the main targets for immunotherapy, and more immune checkpoints and related inhibitors must be identified and employed in the management of malignancy. Furthermore, the current study observed significant differences in immune checkpoints between high- and low-risk ESCC patients. A recent study has confirmed that BTLA can inhibit T cell activity and facilitate tumor evasion, and high expression of BTLA is closely associated with poor prognosis in solid tumors [62]. Tang et al. reported that suppression of HVEM, a ligand for BTLA, inhibited the proliferation of renal cancer cells and slowed tumor growth in vivo [63]. It may be considered that a combination of anti-PD1/PDL1 and anti-BTLA in immunotherapy may further improve tumor control. CD27 is another promising ICI. As a key T cell costimulatory receptor, CD27 participates in the proliferation and differentiation of T cells and plays an important role in immunosuppression [64]. Muth et al. reported that inhibitor of CD27 on regulatory T cells and the application of PD-1 inhibitors synergistically improve the infiltration and functionality of CD8+ T cells in the tumor microenvironment [65]. Thus, CD27 may serve as an effective target for anticancer immunotherapy in combination with PD-1/PD-L1 ICIs.

The current study has several limitations. First of all, cluster identification, establishment of the prognostic model, and validation were performed using retrospective data obtained from a public database. Our own independent validation database collected from our clinical situations has a small sample sizes. Hence, large-scale database should be collected and analyzed to test the effectiveness of our prediction model in clinical scenarios. In addition, the public databases provided limited data regarding significant clinical characteristics, which might have reduced the efficiency of the current prediction model regardless of the fact that the present study endeavored to minimize the risk using multivariate Cox regression analyses. Second, only a limited number of FRGs were included in the present study. The possibility that additional ferroptosis regulators may have been identified is undeniable, owing to the rapid emergence of new studies on ferroptosis. Third, the correlation between risk and biological function of FRLs in ESCC warrant further experimental investigations.

## 5. Conclusions

In summary, the current study observed that FRLs could be used to classify patients with ESCC according to their respective clinical and molecular features. The novel prognostic model with ten FRLs could independently predict the risk associated with the survival of patients with ESCC in the derivation and validation cohorts, which indicated a strong predictive value. The potential mechanisms associated with FRLs and their biological functions in ESCC remain unclear and warrant further research.

## Data Availability

The data used to support the findings of this study are available from the corresponding authors upon request.

## Abbreviations

ESCC: Esophageal squamous cell carcinoma
FRLs: Ferroptosis-related long noncoding RNAs
lncRNA: Long noncoding RNA
TIME: Tumor immune microenvironment
ICIs: Immune checkpoint inhibitors
LASSO: Least absolute shrinkage and selection operator
qRT-PCR: Real-time quantitative polymerase chain reaction
FRGs: Ferroptosis-related genes
OS: Overall survival
DELs: Differentially expressed lncRNAs
GO: Gene Ontology
KEGG: Kyoto Encyclopedia of Genes and Genomes
ssGSEA: Single-sample gene set enrichment analysis
c-index: Concordance index
ROC: Receiver operating characteristic
GSEA: Gene set enrichment analysis
NEO: Neoantigen
TMB: Tumor mutation burden
DEGs: Differentially expressed genes
CCR: Chemokine receptor
DCs: Dendritic cells
BP: Biological processes
CC: Cell component
MF: Molecular function
PD: Progressive disease
SD: Standard deviation
PR: Partial response
CR: Complete response

## Supplementary Materials

Supplementary 1 Coexpression network analysis of ferroptosis-associated lncRNA and mRNA.

Supplementary 2 Identification of differentially expressed mRNAs among the clusters 1, 2, and 3 in TCGA-ESCC cohort. Volcano plot showing the fold change and statistical significance of the mRNA expression between clusters 1 and 2 (A), 1 and 3 (B), 2 and 3 (C).

Supplementary 3 LASSO coefficient profiles of the expression of candidate FRLs (A). Selection of the penalty parameter (*λ*) in the LASSO model (B).

Supplementary 4 The Kaplan–Meier curves show the ten FRLs significantly associated with overall survival in TCGA-ESCC cohort. SNHG29 (A), RB1-DT (B), MEG3 (C), LINC02269 (D), LINC01970 (E), LOC100507144 (F), FAM13A-AS1 (G), EBLN3P (H), CAHM (I), and APOA1-AS (J).

Supplementary 5 Relationships between the risk score model and important clinical characteristics. Gender (A), Age (B), Grade (C), TNM stage (D), and Cluster group (E). The differences were compared using the Wilcoxon test.

Supplementary 6 Stratified survival analysis of the patients with ESCC in TCGA dataset. The patients were assigned to different subgroups according to the clinicopathological risk factors. Age ≤ 65 years (A), male patients (B), patients with grades 3-4 of the disease (C), patients with stage III and IV of the disease (D).

Supplementary 7 Nomogram model was tested by proportional hazard assumption with a global test of the Schoenfeld residuals. Schoenfeld residual test for stage (A). Schoenfeld residual test for risk score (B).

Supplementary 8 GSEA of high- and low-risk groups on the basis of the FRL prognostic signature. GSEA results show significant upregulation of the VEGF (A), IL-17 (B), and ErbB (C) signaling pathway among the ESCC patients in the high-risk group, and Wnt signaling pathway (D) and ferroptosis (E) in the low-risk group.

Supplementary 9 Clinical characteristics of the enrolled patients in TCGA, GSE53624, GSE53625, and an independent validation cohort.

Supplementary 10 The primer sequences of ten FRLS.

Supplementary 11 The likelihood ratio test between different models.
