# Supplementary figures and images for: Ferroptosis-Related lncRNA Signature Correlates with the Prognosis, Tumor Microenvironment, and Therapeutic Sensitivity of Esophageal Squamous Cell Carcinoma
Source: Oxid Med Cell Longev. 2022 Jul 16;2022:7465880. doi: 10.1155/2022/7465880 (PMC9315452; doi:10.1155/2022/7465880)

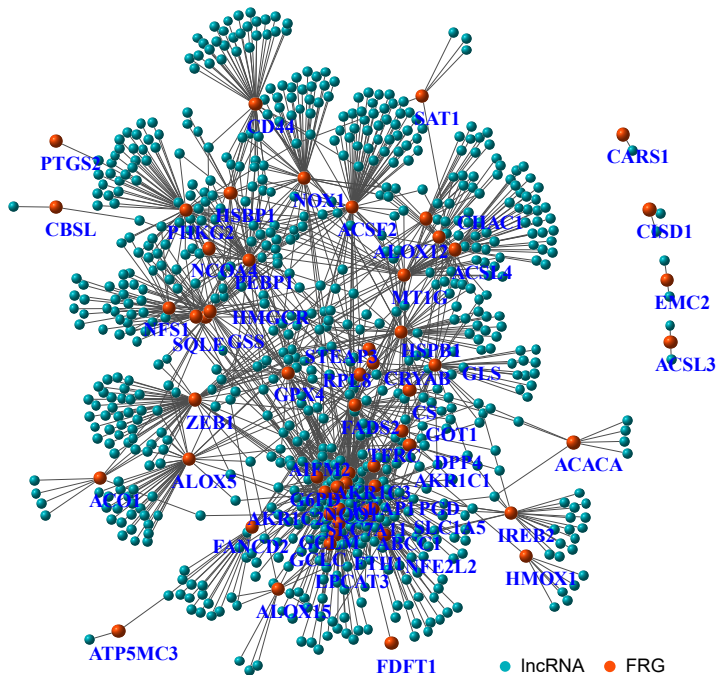

Supplement: Supplementary 1 — Coexpression network analysis of ferroptosis-associated lncRNA and mRNA. [file 7465880.f1.pdf]

A

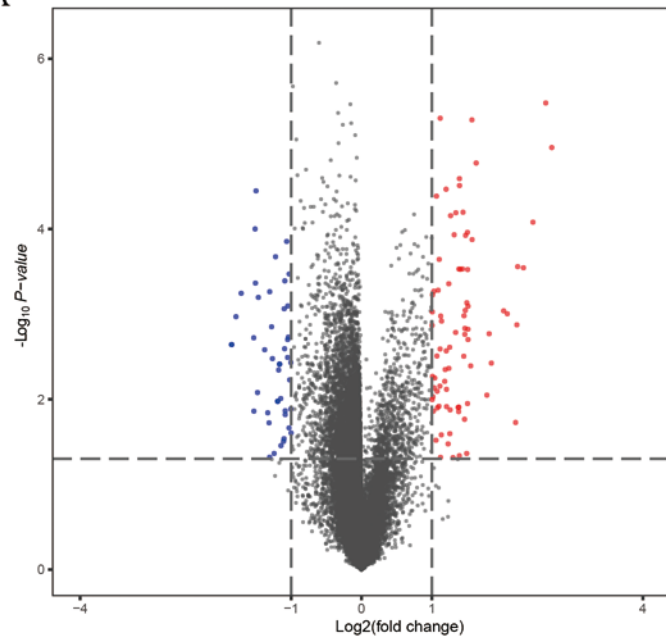

B

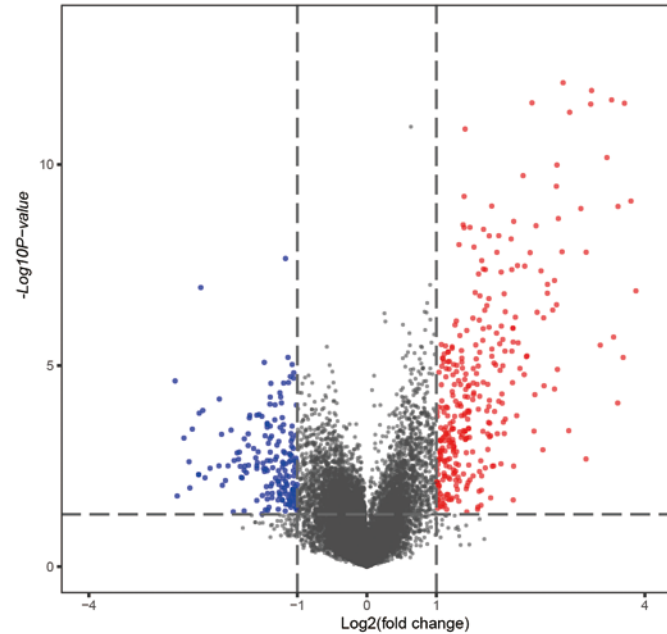

C

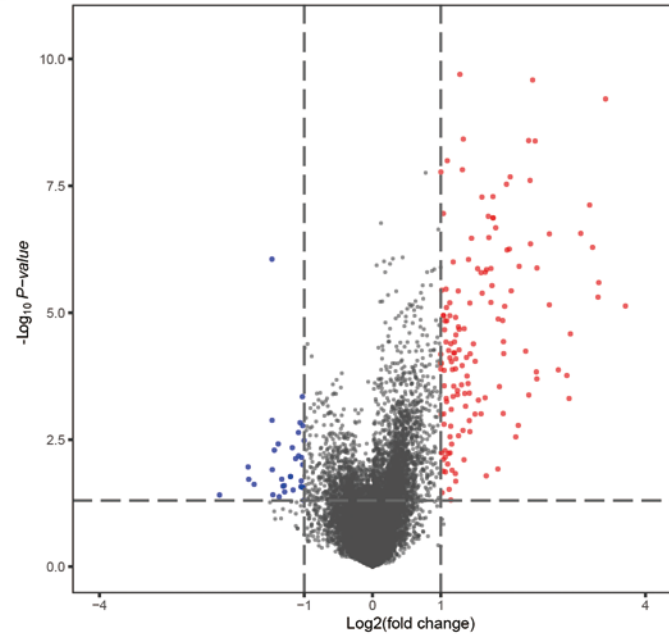

Supplement: Supplementary 2 — Identification of differentially expressed mRNAs among the clusters 1, 2, and 3 in TCGA-ESCC cohort. Volcano plot showing the fold change and statistical significance of the mRNA expression between clusters 1 and 2 (A), 1 and 3 (B), 2 and 3 (C). [file 7465880.f2.pdf]

Group — High Risk — Low Risk

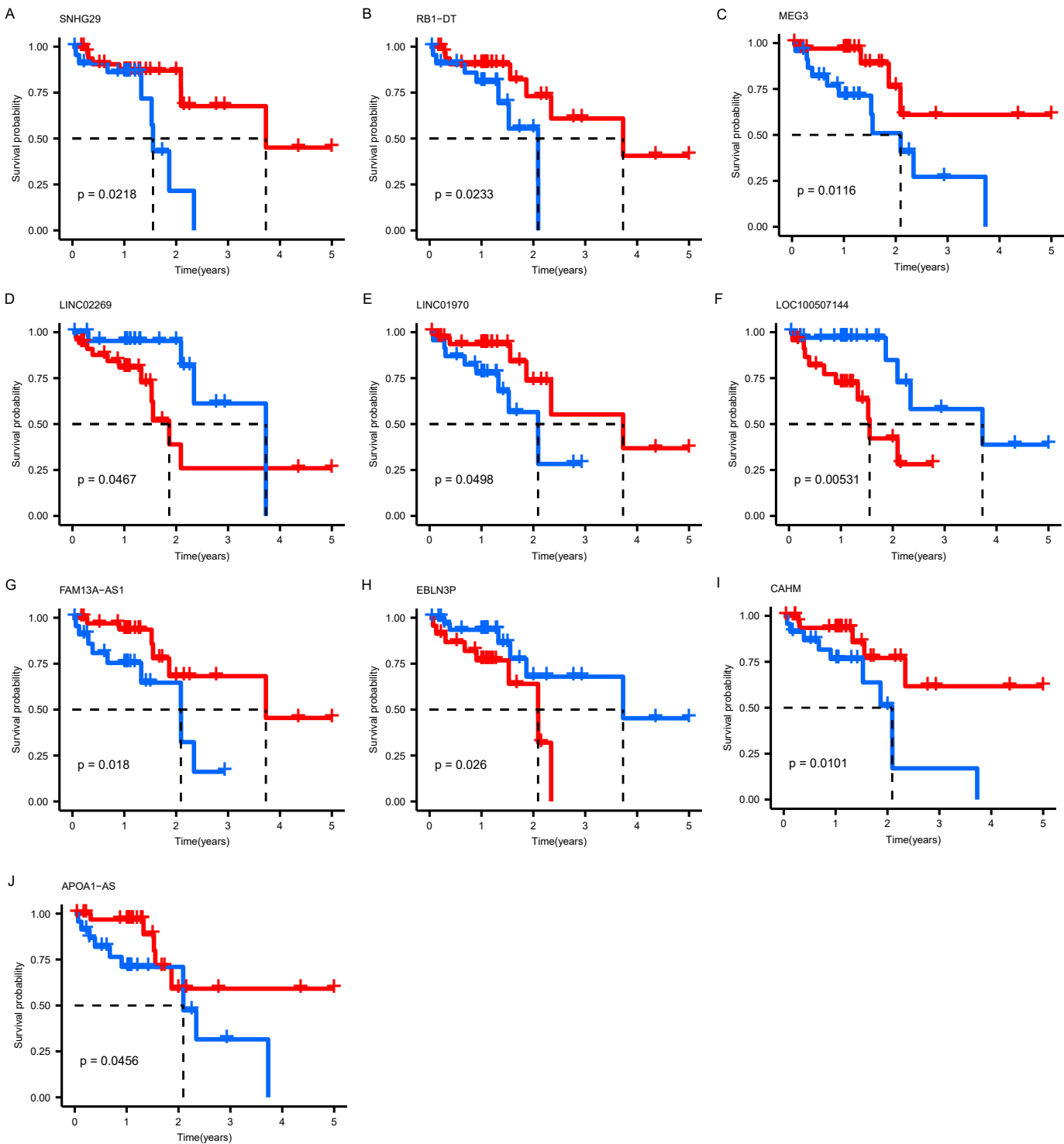

Supplement: Supplementary 4 — The Kaplan–Meier curves show the ten FRLs significantly associated with overall survival in TCGA-ESCC cohort. SNHG29 (A), RB1-DT (B), MEG3 (C), LINC02269 (D), LINC01970 (E), LOC100507144 (F), FAM13A-AS1 (G), EBLN3P (H), CAHM (I), and APOA1-AS (J). [file 7465880.f4.pdf]

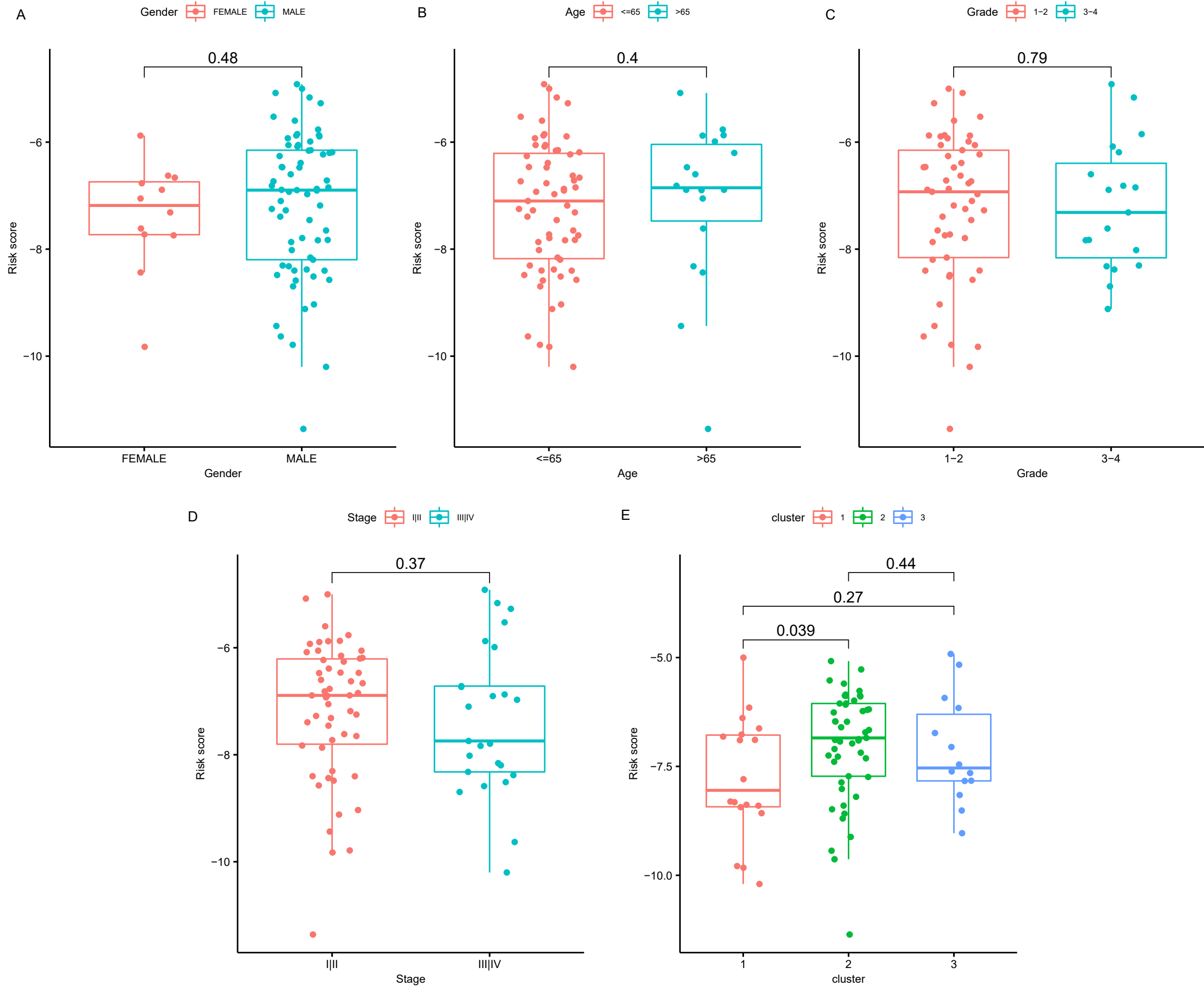

Supplement: Supplementary 5 — Relationships between the risk score model and important clinical characteristics. Gender (A), Age (B), Grade (C), TNM stage (D), and Cluster group (E). The differences were compared using the Wilcoxon test. [file 7465880.f5.pdf]

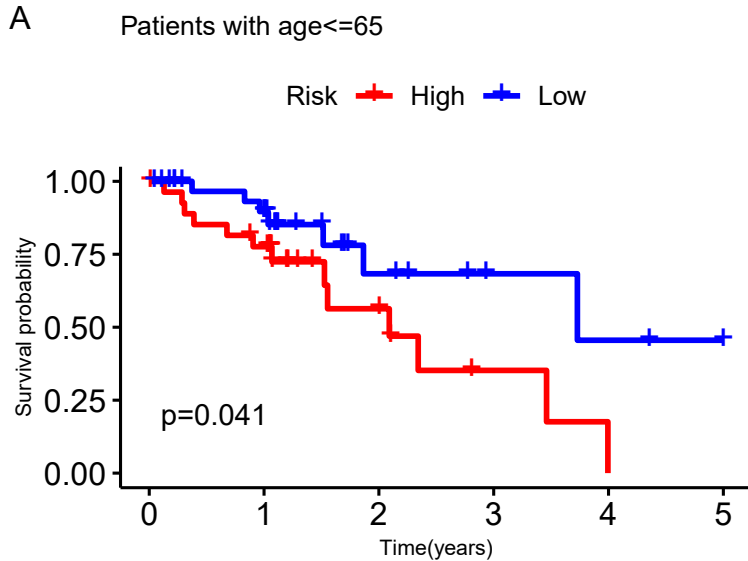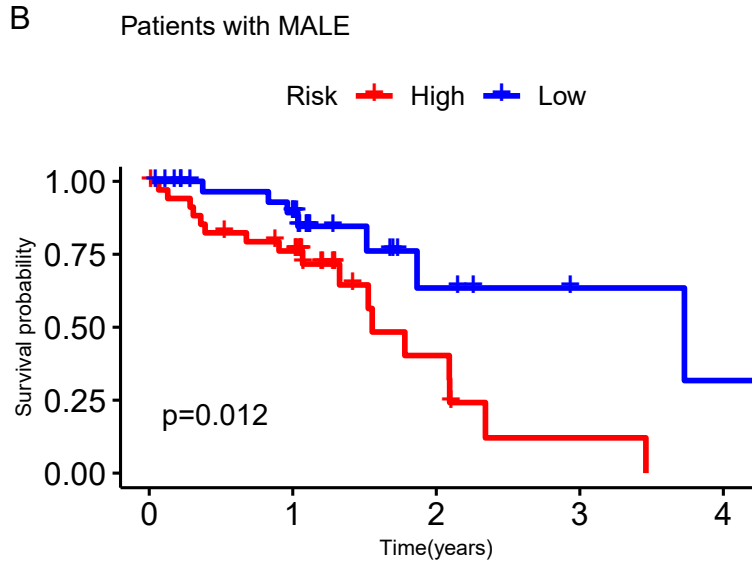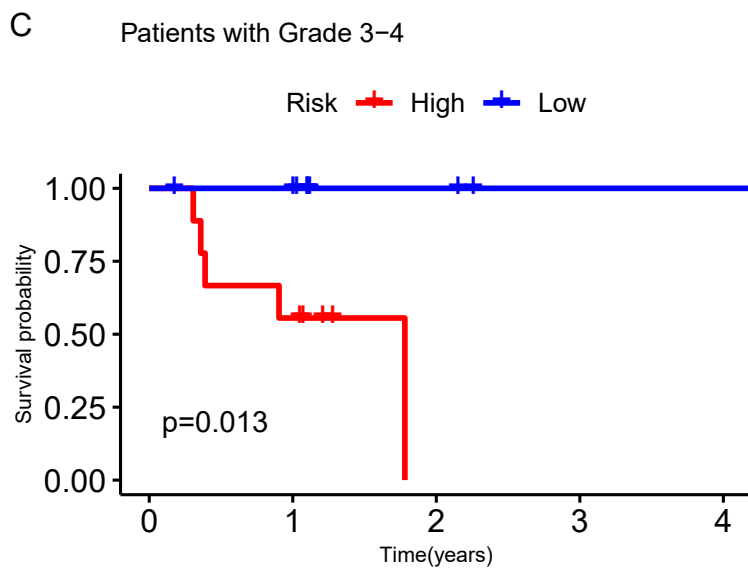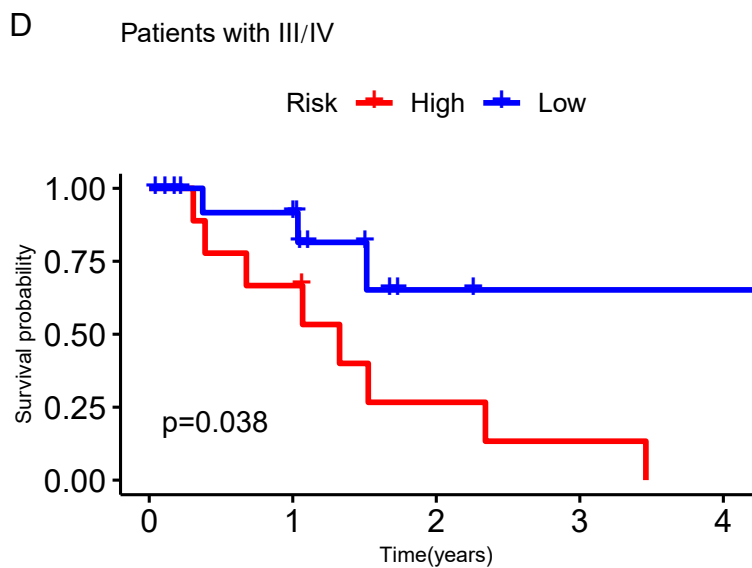

Supplement: Supplementary 6 — Stratified survival analysis of the patients with ESCC in TCGA dataset. The patients were assigned to different subgroups according to the clinicopathological risk factors. Age ≤ 65 years (A), male patients (B), patients with grades 3-4 of the disease (C), patients with stage III and IV of the disease (D). [file 7465880.f6.pdf]

Global Schoenfeld Test p: 0.2873

A  
Schoenfeld Individual Test p: 0.3266

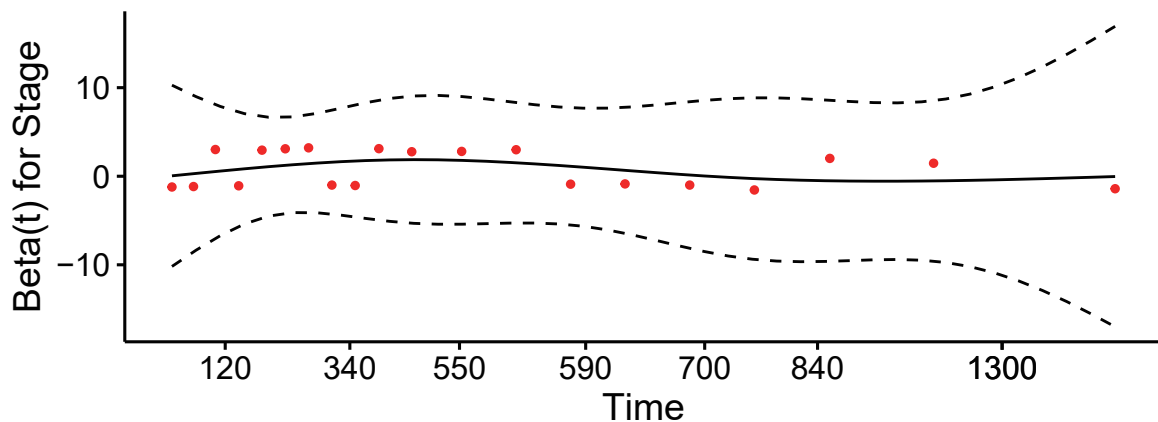

B  
Schoenfeld Individual Test p: 0.2701

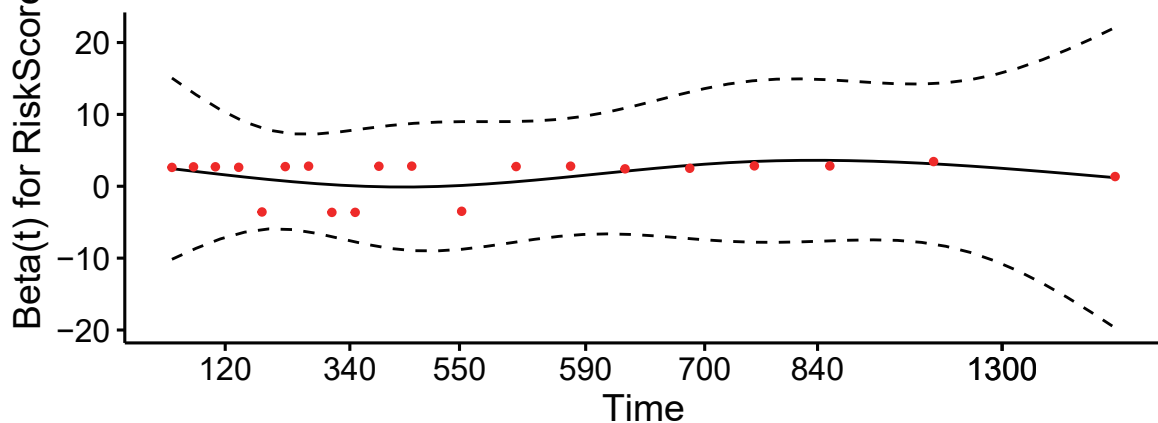

Supplement: Supplementary 7 — Nomogram model was tested by proportional hazard assumption with a global test of the Schoenfeld residuals. Schoenfeld residual test for stage (A). Schoenfeld residual test for risk score (B). [file 7465880.f7.pdf]

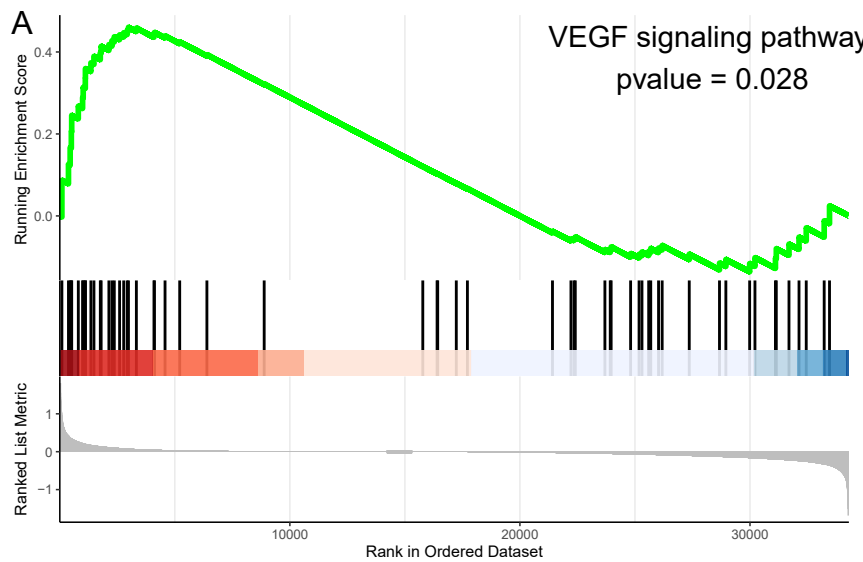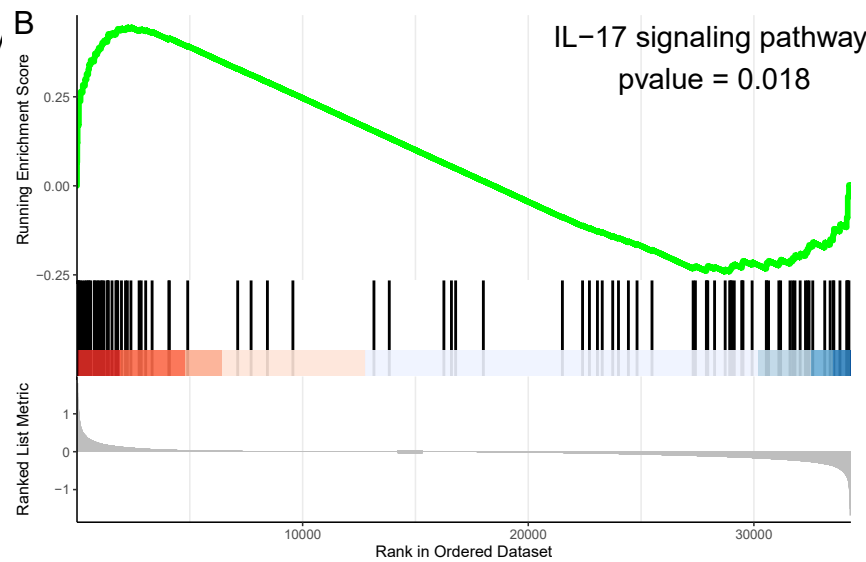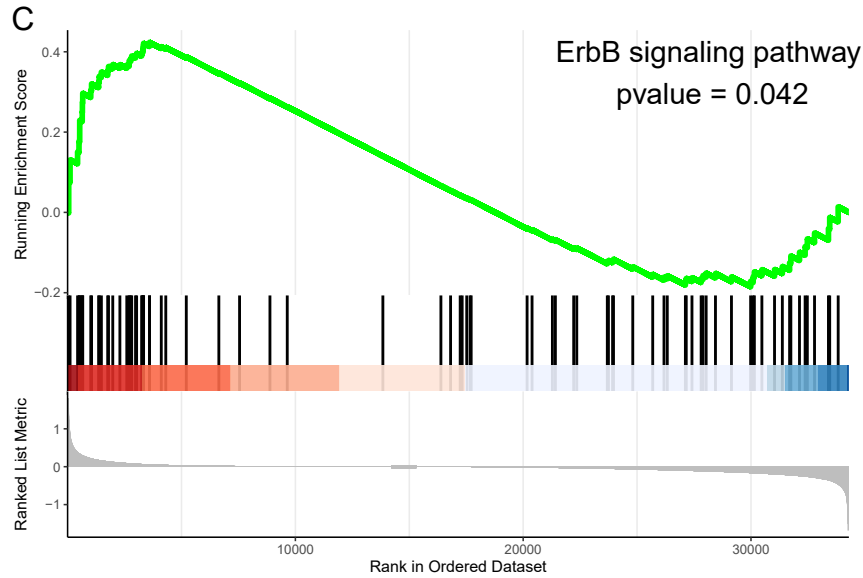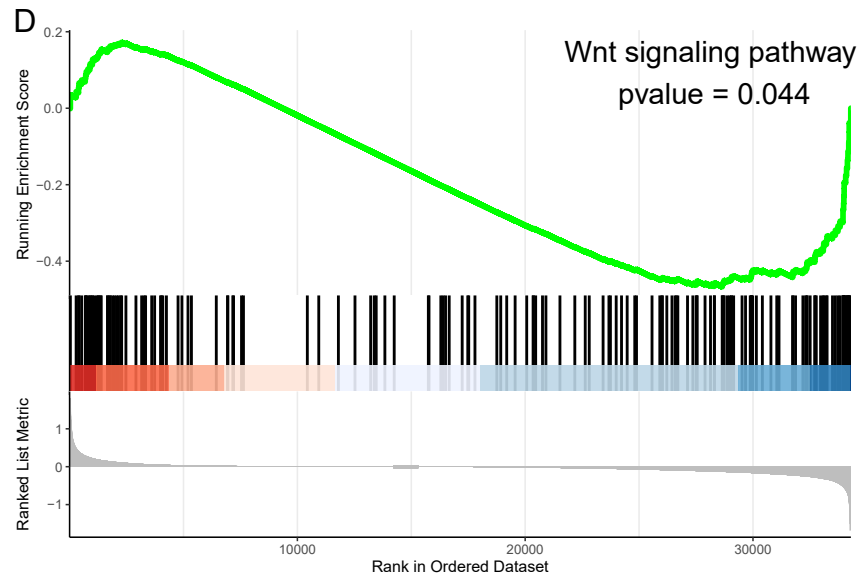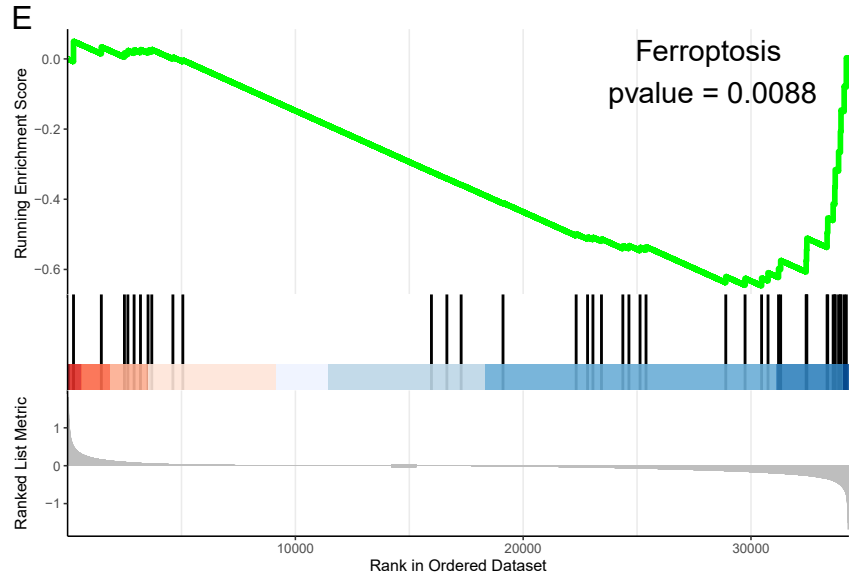

Supplement: Supplementary 8 — GSEA of high- and low-risk groups on the basis of the FRL prognostic signature. GSEA results show significant upregulation of the VEGF (A), IL-17 (B), and ErbB (C) signaling pathway among the ESCC patients in the high-risk group, and Wnt signaling pathway (D) and ferroptosis (E) in the low-risk group. [file 7465880.f8.pdf]
