# Supplementary material for: Ferroptosis-Related lncRNA Signature Correlates with the Prognosis, Tumor Microenvironment, and Therapeutic Sensitivity of Esophageal Squamous Cell Carcinoma
Source: Oxid Med Cell Longev. 2022 Jul 16;2022:7465880. doi: 10.1155/2022/7465880 (PMC9315452; doi:10.1155/2022/7465880)
Supplement: Supplementary 9 — Clinical characteristics of the enrolled patients in TCGA, GSE53624, GSE53625, and an independent validation cohort. [file 7465880.f9.docx]

Table S1. Clinical characteristics of the enrolled patients in TCGA, GSE53624, GSE53625 and an indepndent validation cohort.

|  | Training Set (TCGA-ESCC) | Validation Set (GSE53624) | Validation Set  (GSE53625) | Indepndent Validation Cohort |
| --- | --- | --- | --- | --- |
|  | n=81 | n=119 | n=179 | n=35 |
| Age (years) |  |  |  |  |
| ≤ 65 | 63 (77.78%) | 88 (73.95%) | 130 (72.63%) | 23 (65.7%) |
| ＞65 | 18 (22.22%) | 31 (26.05%) | 49 (27.37%) | 12 (34.3%) |
| Gender |  |  |  |  |
| Male | 69 (85.19%) | 98 (82.35%) | 146 (81.56%) | 26 (74.3%) |
| Female | 12 (14.81%) | 21 (17.65%) | 33 (18.44%) | 9 (25.7%) |
| Tumor stage |  |  |  |  |
| T1 | 8 (9.88%) | 8 (6.72%) | 12 (6.70%) | 5 (14.3%) |
| T2 | 28 (34.57%) | 20 (16.81%) | 27 (15.08%) | 6 (17.1%) |
| T3 | 40 (49.38%) | 62 (52.10%) | 110 (61.45%) | 14 (40.0%) |
| T4 | 5 (6.17%) | 29 (24.37%) | 30 (16.76%) | 10 (28.6%) |
| N stage |  |  |  |  |
| N- | 46 (56.79%) | 54 (45.38%) | 83 (46.37%) | 18 (51.4%) |
| N+ | 35 (43.21%) | 65 (54.62%) | 96 (53.63%) | 17 (48.6%) |
| TNM stage |  |  |  |  |
| I-II | 42 (51.85%) | 53 (44.54%) | 87 (48.60%) | 11 (31.4%) |
| III-IV | 39 (48.15%) | 66 (55.46%) | 92 (51.40%) | 24 (68.6%) |
| Grade |  |  |  |  |
| 1-2 | 58 (71.60%) | 87 (73.11%) | 130 (72.63%) | 21 (60%) |
| 3-4 | 23 (28.40%) | 32 (26.89%) | 49 (27.37%) | 14 (40%) |
| Vital status |  |  |  |  |
| Dead | 25 (30.86%) | 73 (61.34%) | 106 (59.22%) | 12 (34.3%) |
| Alive | 56 (69.14%) | 46 (38.66%) | 73 (40.78%) | 23 (65.7%) |

*Abbreviations*: TNM: tumor node metastasis.
