# Supplementary material for: Ferroptosis-Related lncRNA Signature Correlates with the Prognosis, Tumor Microenvironment, and Therapeutic Sensitivity of Esophageal Squamous Cell Carcinoma
Source: Oxid Med Cell Longev. 2022 Jul 16;2022:7465880. doi: 10.1155/2022/7465880 (PMC9315452; doi:10.1155/2022/7465880)
Supplement: Supplementary 10 — The primer sequences of ten FRLS. [file 7465880.f10.docx]

Table S2. The primer sequences of ten FRLS

| LncRNA | Sequence |
| --- | --- |
| hMEG3-QPCR-F | CATCCGTCCACCTCCTTGTCTTC |
| hMEG3-QPCR-R | GTCCTCTTCATCCTTTGCCATCC |
| hSNHG29-F | CTGGCGCACATAAGGTGTGA |
| hSNHG29-R | CAGGCTCCTGGCTCCAATAC |
| hRB1-DT-F | TTGCAAAGTCGGCCAAAAC |
| hRB1-DT-R | GCAGTCTGGACACTTGGTAC |
| hLOC100507144-F | AGGACTGATTCGACCCCGTA |
| hLOC100507144-R | ATGGAGGTGTATTGCTGCCC |
| hLINC02269-F | GTGCTTCCAGGGTAAGGCAT |
| hLINC02269-R | TCGCAGGAATGAAAGGCTGT |
| hLINC01970-F | GGATCGGAAACAGCCGAGTC |
| hLINC01970-R | TCTTGCATCACTGGTACGGC |
| hFAM13A-AS1-F | ATGCCCCTCACATGGACTTG |
| hFAM13A-AS1-R | CCACTCGGCAACACTGATTC |
| hEBLN3P-F | TACGCGTTTTGGTCCCTGTT |
| hEBLN3P-R | GCCACTTGGCTCAAAAGACTG |
| hCAHM-F | GTCGTGCTCGAAATGCTTCC |
| hCAHM-R | GCTTTTCAGACTACGCGCAC |
| hAPOA1-AS-F | ATGCTGGTCACTTCAGTCCC |
| hAPOA1-AS-R | AGGGGATTGGTTATGAGGCT |
| hbactin-QPCR-F | CTGGGACGACATGGAGAAAATC |
| hbactin-QPCR-R | CCCCTCGTAGATGGGCACA |
