# Supplementary material for: Ferroptosis-Related lncRNA Signature Correlates with the Prognosis, Tumor Microenvironment, and Therapeutic Sensitivity of Esophageal Squamous Cell Carcinoma
Source: Oxid Med Cell Longev. 2022 Jul 16;2022:7465880. doi: 10.1155/2022/7465880 (PMC9315452; doi:10.1155/2022/7465880)
Supplement: Supplementary 11 — The likelihood ratio test between different models. [file 7465880.f11.docx]

Table S3. Likelihood ratio test between different models.

| Nomogram model vs. Stage model | | | |
| --- | --- | --- | --- |
| Likelihood ratio | Chisq | Df | *P* |
| -60.165 |  |  |  |
| -64.092 | 7.853 | 1 | 0.005 |
|  | | | |
| Nomogram model vs. RiskScore model | | | |
| Likelihood ratio | Chisq | Df | *P* |
| -60.165 |  |  |  |
| -61.689 | 3.047 | 1 | 0.041 |
