# Supplementary material for: Ferroptosis-Related lncRNA Signature Correlates with the Prognosis, Tumor Microenvironment, and Therapeutic Sensitivity of Esophageal Squamous Cell Carcinoma
Source: Oxid Med Cell Longev. 2022 Jul 16;2022:7465880. doi: 10.1155/2022/7465880 (PMC9315452; doi:10.1155/2022/7465880)
Supplement: Supplementary 3 — LASSO coefficient profiles of the expression of candidate FRLs (A). Selection of the penalty parameter (λ) in the LASSO model (B). [file 7465880.f3.pdf]

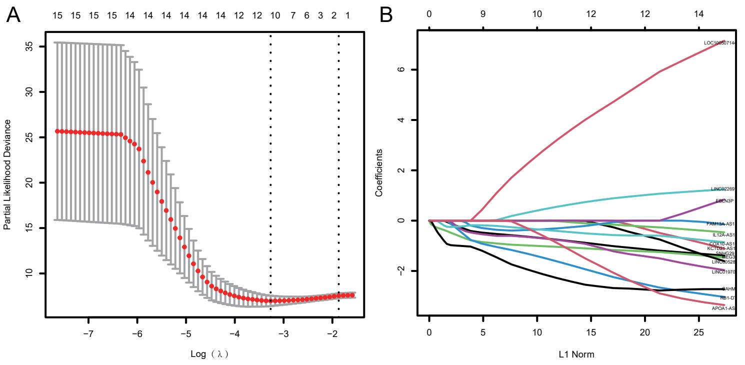
A
B
Partial Likelihood Deviance
Log（λ）
Coefficients
L1 Norm
LOC100507144
LINC02269
EBLN3P
FAM13A-AS1
IL12A-AS1
COX10-AS1
KCTD21-AS1
SNHG29
MEG3
LINC00528
LINC01970
SAHM
RB1-DT
APOA1-AS
